# Association between the liver fat score (LFS) and cardiovascular diseases in the national health and nutrition examination survey 1999–2016

**DOI:** 10.1080/07853890.2021.1943514

**Published:** 2021-06-29

**Authors:** Chun-On Lee, Hang-Long Li, Man-Fung Tsoi, Ching-Lung Cheung, Bernard Man Yung Cheung

**Affiliations:** a Department of Medicine, Division of Clinical Pharmacology and Therapeutics, The University of Hong Kong, Hong Kong, China;; b State Key Laboratory of Pharmaceutical Biotechnology, The University of Hong Kong, Hong Kong, China;; c Department of Pharmacology and Pharmacy, The University of Hong Kong, Hong Kong, China;; d Institute of Cardiovascular Science and Medicine, The University of Hong Kong, Hong Kong, China

**Keywords:** Cardiovascular disease prevention, metabolic syndrome, non-alcoholic fatty liver disease score, liver fat score

## Abstract

**Background:**

The liver fat score (LFS) has been proposed to be a simple non-invasive marker of non-alcoholic fatty liver disease (NAFLD), which is highly prevalent in the general population. We tested its association with cardiovascular diseases (CVDs) and prognosis.

**Methods:**

17,244 adult participants from the National Health and Nutrition Examination Survey 1999–2016 were included. LFS is calculated from variables including serum aspartate transaminase/alanine transaminase (AST/ALT) ratio, fasting serum aspartate transaminase (AST) level, fasting serum insulin level, presence of metabolic syndrome and diabetes mellitus. In cross-sectional analysis, logistic regression was used to examine the association of the LFS with coronary heart disease (CHD), myocardial infarction (MI), congestive heart failure (CHF), stroke and angina pectoris. Mortality during follow-up was analysed using Cox proportional hazard regression.

**Results:**

LFS was associated with CHD (adjusted odds ratio [OR]: 1.09 per standard deviation [SD], 95% confidence interval [95% CI]: 1.03–1.15) (*p* = .003), CHF (1.11, 1.04–1.18) (*p* = .003) and angina pectoris (1.08, 1.02–1.13) (*p* = .005). LFS was not associated with MI or stroke, but was associated with increased all-cause and cardiovascular mortality with hazard ratios (HRs) of 1.10 (95% CI: 1.07–1.13) (*p* < .001) and 1.12 (95% CI: 1.06–1.17) (*p* < .001), respectively.

**Conclusions:**

NAFLD is usually asymptomatic, but this large study of a large general population shows that LFS is associated with CHD, CHF, angina pectoris, cardiovascular and all-cause mortality. Determining the LFS is worthwhile, as it identifies people with NAFLD, who may also be at increased cardiovascular risk.

**Key Messages**
- Liver fat score (LFS), a non-invasive marker of non-alcoholic fatty liver disease (NAFLD), is associated with coronary heart disease (CHD), congestive heart failure (CHF) and angina.
- LFS is also associated with increased cardiovascular and all-cause mortality.
- Determining the LFS is worthwhile as it identifies people with NAFLD as well as increased cardiovascular risk.

## 1. Introduction

Non-alcoholic fatty liver disease (NAFLD) includes a spectrum of progressive liver abnormalities ranging from simple steatosis, non-alcoholic steatohepatitis (NASH), advanced fibrosis to cirrhosis with varied prognosis. The prevalence of NAFLD is 25% in the United States (US) [1]. NASH has been the fastest growing indication for liver transplantation in patients with hepatocellular carcinoma in the US [2]. With the increasing prevalence of obesity, we anticipate a continuous rising trend in NAFLD [1]. Therefore, NAFLD has become an important public health issue; there is a pressing need to identify the disease.

There is a continuum from central obesity and metabolic syndrome to atherosclerosis, ischaemic heart disease and heart failure [3]. NAFLD is closely associated with the metabolic syndrome [4], and may be an early stage in this process. Several studies showed increased cardiovascular disease (CVD) events in NAFLD patients with type 1 and type 2 diabetes mellitus [5,6]. Furthermore, NAFLD is inextricably linked to cardiovascular diseases (CVDs)cardiovascular diseases (CVDs) [7–10]. There is an association between elevation of alanine aminotransferase (ALT), a biomarker of NAFLD and CVDs [11]. Besides ALT, elevations in other liver enzymes are also associated with generalized inflammation, hypertension and vascular disease [12–16]. NAFLD is also associated with higher cardiovascular mortality (CV mortality) and all-cause mortality [10,17–20]. Therefore, identifying NAFLD also helps to identify patients at increased CVD risk at the same time.

Liver biopsy is considered as the gold standard for the diagnosis of NAFLD. However, it is an invasive procedure and therefore selectively performed in patients with suspected advanced disease. Liver ultrasonography is more widely used for screening for NAFLD in clinical practice and research. However, it is operator dependent and has low sensitivity in patients with mild steatosis. New quantitative imaging modalities, e.g. proton magnetic resonance spectroscopy ((1)H-MRS), are costly and resource-intensive.

In recent years, five simple scoring systems based on simple clinical, anthropometric and laboratory data have been developed for non-invasive NAFLD detection: (i) NAFLD liver fat score (LFS) [21]^;^ (ii) fatty liver index (FLI) [22]; (ii) hepatic steatosis index (HSI) [23]; (iv) lipid accumulation product (LAP) [24]; (v) SteatoTest [25]. Our previous study validating the first four aforementioned NAFLD scores revealed that LFS performed best in identifying ultrasonography-diagnosed NAFLD with high sensitivity and specificity [17]. Whereas FLI, HSI and LAP were derived from ultrasonography, LFS was derived from proton magnetic resonance spectroscopy, which is the most quantitative and sensitive currently available imaging method. LFS may therefore be useful for large-scale NAFLD screening in the population. In addition, there are also the Hepamet fibrosis score (HFS) [26], fibrosis-4 index (FIB4) [27] and non-alcoholic fatty liver disease fibrosis score (NFS) [28], derived and validated in studies with liver biopsy, that predict the severity of fibrosis in NAFLD patients [29–31].

The utility of NAFLD screening is more than the detection of NAFLD. As it is associated with CVDs, the presence of NAFLD should prompt the search for CVDs. We therefore analysed the association of NAFLD, mainly LFS, with five cardiovascular conditions, namely, coronary heart disease (CHD), myocardial infarction (MI), congestive heart failure (CHF), stroke and angina pectoris, using data from the US National Health and Nutrition Examination Survey (NHANES) 1999–2016 [32]. We also studied the association of NAFLD scores with all-cause mortality and CV mortality.

### 2. Materials and methods

US NHANES is a continuous national survey conducted by the National Centre for Health Statistics of the Centres for Disease Control and Prevention [33]. Data are released in every 2-year cycle. Each participant represents about 50,000 US citizens. All participants gave informed consent before participation and ethics approval of the study was obtained from the Research Ethics Review Board at the National Centre for Health Statistics. Detailed study methodology and protocols are given on its website [33].

We studied non-pregnant participants aged 20 or above in NHANES 1999–2016 for cross-sectional analysis. For inclusion in the analysis, the levels of blood-based biomarkers for all non-invasive score formulae had to be available. To avoid the confounding effect on the diagnosis of NAFLD, participants with excessive alcohol use (defined as > 21 drinks per week for men and > 14 drinks per week for women) and viral hepatitis (defined as laboratory findings of positive serum hepatitis B surface antigen; or positive serum hepatitis C antibody; or positive serum hepatitis C virus ribonucleic acid if hepatitis C antibody was not applicable) were excluded. We included a total of 17,244 participants in this study.

Venous blood samples were obtained in Mobile Examination Centres according to a standard protocol. Several laboratory variables used for inclusion or exclusion criteria and involved in the computation of non-invasive NAFLD scores, including hepatitis B surface antigen, hepatitis C antibody or virus ribonucleic acid, liver function test, lipid profile, fasting glucose, fasting insulin, glycosylated haemoglobin, albumin and platelet count, were measured. Detailed laboratory procedures and protocol are provided on the NHANES website [33].

### 2.1. Variables of interest

Baseline characteristics of participants including age, ethnicity, education level, smoking status and alcohol consumption were obtained by questionnaire [33]. CVD events, including CHD, MI, CHF, stroke and angina pectoris, were recorded based on self-reported medical history. Composite CVD events, comprising CHD, MI, CHF and stroke, were also studied. As angina pectoris is a symptom complex rather than a disease entity, we did not include it in the composite CVD events. Previous diagnoses of CVDs were made by doctors or other healthcare professionals. Anthropometric measurements were done in Mobile Examination Centres according to a standard protocol [33].

LFS uses serum aspartate transaminase/alanine transaminase (AST/ALT) ratio, fasting serum aspartate transaminase (AST) level, fasting serum insulin level, presence of metabolic syndrome and diabetes mellitus to calculate the score [21]. Besides LFS, we studied other NAFLD markers (FLI, HSI, LAP, HFS, FIB4 and NFS). We did not evaluate the SteatoTest as its scoring system is not disclosed for commercial reasons. Variables needed to calculate the scores differ among the scoring systems. The formulae of the seven scores are listed in Additional Table 1 [21–28].

### 2.2. Mortality follow-up

National Centre for Health Statistics (NCHS) Public-use Linked Mortality Files match NHANES data with the National Death Index (NDI) death certificate records [34,35]. Cause-specific deaths were coded using the Underlying Cause of Death. CV death was defined by the International Classification Disease 10th Edition I00–I09, I11, I13, I20–I51, I60–I69 (I60–I69 for NHANES 1999–2006 only) [35]. Length of follow-up was from the time from the NHANES interview date to the end of follow-up (date of death or 31 December 2015). After excluding participants with missing mortality data, 15,151 participants were included in the prospective analysis of mortality.

### 2.3. Definitions of diabetes mellitus, hypertension and metabolic syndrome

We defined diabetes mellitus as fasting glucose ≥126 mg/dL, glycosylated haemoglobin ≥6.5% or previous diagnosis by a doctor. A previous diagnosis by a doctor was determined by the question “have you/has survey participant ever been told by a doctor or other health professional that you/she/he had diabetes or sugar diabetes”? [33]

Blood pressure was measured three times using a mercury sphygmomanometer (Baumanometer^®^, W. A. Baum Co., Inc., Copiague, NY) after the participants had rested quietly in a seated position for 5 min. Up to four readings could be obtained if an earlier reading was interrupted or incomplete. All measurements were done in mobile examination centres by trained and certified personnel [33]. We regarded participants as hypertensive if at least three of the blood pressure measurements on the day of examination were ≥130 mmHg for systolic measurement or ≥80 mmHg for diastolic measurement; or if they were previously diagnosed by a doctor. A previous diagnosis by a doctor was determined by the question “have you/has survey participant ever been told by a doctor or other health professional that you/she/he had hypertension, also called high blood pressure”? [33]

Participants were considered as having metabolic syndrome if they met at least three of the following five conditions: (i) waist circumference ≥1.02 m in men or ≥0.88 m in women of European descent; (ii) systolic blood pressure ≥130 mmHg, diastolic blood pressure ≥85 mmHg or being prescribed anti-hypertensive drugs; (iii) serum triglycerides level ≥1.70 mmol/L or being prescribed drugs for elevated triglycerides; (iv) serum high-density lipoprotein cholesterol level <1.00 mmol/L in men or <1.30 mmol/L in women or being prescribed drugs for reduced high-density lipoprotein cholesterol; and (v) fasting plasma glucose level ≥5.6 mmol/L (≥100 mg/dL) or being prescribed anti-diabetic drugs [36].

### 2.4. Statistical analysis

We analysed the data using SPSS complex sample module version 24.0 (IBM Corp, Armonk, NY) and the “survey” package in R version 3.6.3 (Vienna, Austria). Two-year mobile examination weights were added to account for unequal probabilities of selection, non-response bias and oversampling; the 4-year mobile examination weights, provided only in 1999–2002, were multiplied by 2. Continuous variables were expressed as weighted means with standard errors or with 95% confidence interval (95% CI) while categorical variables were expressed as the estimated population with weighted percentages. Multiple logistic regression analysis was used to study the association of every increase in standard deviation (SD) of non-invasive NAFLD scores and CVDs, adjusted for age, gender and ethnicity in model 1, and further adjusted for high-density lipoprotein cholesterol level, smoking status, statin use and aspirin use in model 2. To further assess the utility of LFS as a marker of CVD outcomes, the areas under the “receiver operating curve” (AUC) were calculated. Cox proportional hazard regression model was used to assess the association of NAFLD scores and mortality.

## 3. Results

In this study, we included 17,244 participants from NHANES 1999–2016. Characteristics of the included participants are summarized in Table 1. The mean ± SD of LFS was −0.32 ± 2.68. The means of other non-invasive NAFLD scores are summarized in Additional Table 2.

**Table 1.** Demographics of participants included in this analysis.

Year	1999–2000	2001–2002	2003–2004	2005–2006	2007–2008
%	9.7%	10.9%	9.6%	9.8%	11.1%
Age	46.32 ± 0.77	46.47 ± 0.95	46.73 ± 0.64	47.75 ± 0.94	47.27 ± 0.69
Female (%)	3,568,529 (50.3%)	4,134,820 (51.8%)	3,568,730 (50.7%)	3,632,058 (50.3%)	4,195,864 (51.5%)
Ethnicity					
Mexican American (%)	499,167 (7.0%)	543,553 (6.8%)	520,583 (7.4%)	519,397 (7.2%)	681,875 (8.4%)
Other Hispanic (%)	643,007 (9.1%)	323,712 (4.1%)	191,713 (2.7%)	248,163 (3.4%)	391,425 (4.8%)
Non-Hispanic White (%)	5,009,423 (70.6%)	6,104,945 (76.5%)	5,253,653 (74.6%)	5,252,318 (72.7%)	5,755,268 (70.6%)
Non-Hispanic Black (%)	7,348,633 (10.0%)	7,137,575 (9.2%)	6,983,431 (9.9%)	8,010,436 (11.1%)	8,623,158 (10.6%)
Others (%)	2,444,089 (3.3%)	2,606,013 (3.4%)	3,768,943 (5.4%)	4,052,493 (5.6%)	4,632,618 (5.7%)
Non-Hispanic Asian (%)	NA	NA	NA	NA	NA
Other races – including multiracial (%)	NA	NA	NA	NA	NA
DM (%)	568,883 (8.0%)	785,935 (9.9%)	753,931 (10.7%)	838,676 (11.6%)	1,083,298 (13.3%)
HTN (%)	3,205,994 (45.2%)	3,746,471 (47.0%)	3,275,651 (46.5%)	3,313,601 (45.9%)	3,582,152 (43.9%)
MetS (%)	3,457,829 (48.8%)	3,928,341 (49.2%)	3,672,281 (52.2%)	3,499,399 (48.4%)	3,955,183 (48.5%)
Medical history					
CHD (%)	191,100 (2.7%)	274,883 (3.4%)	295,719 (4.2%)	244,068 (3.4%)	285,265 (3.5%)
MI (%)	224,513 (3.2%)	243,564 (3.1%)	253,820 (3.6%)	238,828 (3.3%)	291,213 (3.6%)
CHF (%)	161,177 (2.3%)	149,587 (1.9%)	162,254 (2.3%)	172,332 (2.4%)	166,490 (2.0%)
Stroke (%)	185,120 (2.6%)	156,579 (2.0%)	203,454 (2.9%)	217,759 (3.0%)	208,086 (2.6%)
CVD (%)	495,287 (7.0%)	533,208 (6.7%)	583,079 (8.3%)	581,914 (8.1%)	611,120 (7.5%)
Angina pectoris (%)	212,543 (3.0%)	222,889 (2.8%)	211,327 (3.0%)	194,979 (2.7%)	176,868 (2.2%)
Smoker (%)	3,551,128 (50.1%)	3,966,521 (49.7%)	3,511,372 (49.9%)	3,566,274 (49.4%)	3,749,158 (46.0%)
Concomitant medications					
Statin use (%)	533,683 (7.5%)	832,422 (10.4%)	914,377 (13.0%)	1,036,909 (14.3%)	1,323,361 (16.2%)
Aspirin use (%)	8,951 (0.1%)	35,833 (0.4%)	11,784 (0.2%)	51,874 (0.7%)	61,360 (0.8%)
Waist circumference (m)	0.95 ± 0.01	0.96 ± 0.00	0.98 ± 0.01	0.99 ± 0.01	0.98 ± 0.00
BMI (kg/m^2^)	27.23 (26.65–27.83)	27.35 (27.06–27.63)	27.80 (27.47–28.15)	28.14 (27.71–28.58)	27.78 (27.45–28.11)
Serum HDL cholesterol (mmol/L)*	1.23 (1.20–1.26)	1.27 (1.25–1.29)	1.33 (1.31–1.36)	1.37 (1.35–1.39)	1.33 (1.30–1.36)
LFS	−0.34 ± 0.09	−0.39 ± 0.10	−0.57 ± 0.09	−0.51 ± 0.05	−0.36 ± 0.09

Year	2009–2010	2011–2012	2013–2014	2015–2016	*p* Value
%	11.5%	12.1%	12.4%	12.9%	
Age	47.63 ± 0.61	47.94 ± 0.63	47.88 ± 0.67	49.49 ± 0.67	.095
Female (%)	4,394,810 (52%)	4,541,188 (51.2%)	4,655,956 (51.1%)	4,876,675 (51.6%)	.931
Ethnicity					.401
Mexican American (%)	789,900 (9.3%)	708,585 (8%)	812,645 (8.9%)	744,257 (7.9%)	–
Other Hispanic (%)	477,345 (5.6%)	607,225 (6.8%)	520,023 (5.7%)	622,135 (6.6%)	–
Non-Hispanic White (%)	5,837,009 (69.1%)	6,090,826 (68.6%)	6,160,272 (67.7%)	6,289,975 (66.5%)	–
Non-Hispanic Black (%)	8,255,933 (9.8%)	7,889,813 (8.9%)	9,047,094 (9.9%)	9,405,597 (10%)	–
Others (%)	5,214,515 (6.2%)	6,813,612 (7.7%)	7,072,002 (7.8%)	8,554,748 (9.1%)	–
Non-Hispanic Asian (%)	NA	454,475 (5.1%)	511,963 (5.6%)	488,542 (5.2%)	–
Other race – including multiracial (%)	NA	226,886 (2.6%)	195,237 (2.1%)	366,933 (3.9%)	–
DM (%)	1,120,839 (13.3%)	1,139,360 (12.8%)	1,195,194 (13.1%)	1,371,731 (14.5%)	.001
HTN (%)	3,493,293 (41.3%)	4,115,284 (46.4%)	4,146,676 (45.5%)	4,427,392 (46.8%)	.482
MetS (%)	4,108,214 (48.6%)	4,493,982 (50.6%)	4,552,333 (50%)	5,035,541 (53.3%)	.562
Medical history					
CHD (%)	276,123 (3.3%)	337,241 (3.8%)	339,822 (3.7%)	347,613 (3.7%)	.799
MI (%)	282,541 (3.3%)	289,887 (3.3%)	314,526 (3.5%)	293,401 (3.1%)	.997
CHF (%)	140,283 (1.7%)	281,430 (3.2%)	216,742 (2.4%)	185,463 (2.0%)	.188
Stroke (%)	207,266 (2.5%)	245,621 (2.8%)	261,399 (2.9%)	280,421 (3.0%)	.873
CVD (%)	612,615 (7.2%)	746,647 (8.4%)	764,831 (8.4%)	784,556 (8.3%)	.758
Angina pectoris (%)	176,151 (2.1%)	249,881 (2.8%)	163,981 (1.8%)	177,454 (1.9%)	.376
Smoker (%)	3,636,196 (43.0%)	3,789,569 (42.7%)	3,843,987 (42.2%)	4,266,091 (45.1%)	.012
Concomitant medications					
Statin use (%)	1,515,095 (17.9%)	1,719,715 (19.4%)	1,828,662 (20.1%)	1,992,423 (21.1%)	<.001
Aspirin use (%)	132,877 (1.6%)	53,737 (0.6%)	74,092 (0.8%)	32,363 (0.3%)	<.001
Waist circumference (m)	0.98 ± 0.01	0.99 ± 0.01	1.00 ± 0.00	1.01 ± 0.01	<.001
BMI (kg/m^2^)	27.98 (27.58–28.39)	28.20 (27.74–28.67)	28.34 (28.02–28.67)	28.71 (28.07–29.37)	<.001
Serum HDL cholesterol (mmol/L)*	1.34 (1.32–1.36)	1.32 (1.30–1.35)	1.34 (1.31–1.36)	1.38 (1.35–1.41)	<.001
LFS	–0.13 ± 0.06	–0.21 ± 0.10	–0.29 ± 0.11	–0.16 ± 0.09	<.001

Data are expressed as the estimated population (weighted percentage), weighted mean ± standard error or weighted mean (95% confidence interval).

NA: not applicable; DM: diabetes mellitus; HT: hypertension; MetS: metabolic syndrome; CHD: coronary heart disease; MI: myocardial infarction; CHF: congestive heart failure; CVD: composite cardiovascular disease events consisting of CHD, MI, CHF and stroke; m: metre; BMI: body mass index; kg/m^2^: kilogram per square centimetre; HDL: high-density lipoprotein; mmol/L: millimoles per litre; LFS: non-alcoholic fatty liver disease liver fat score.

*Log-transformed variable was used.

The association between CVDs and every increase of one SD in LFS is summarized in Table 2 and Additional Figure 1. LFS was associated with various types of CVD (Table 2). After adjustment for age, gender, ethnicity, high-density lipoprotein cholesterol level, smoking status, statin use and aspirin use, one SD higher in LFS was associated with higher odds of CHD (adjusted odds ratio [OR] per SD change: 1.09 [95% CI: 1.03–1.15]) (*p*=.003), CHF (1.11 [1.04–1.18]) (*p*=.003), composite CVD events (1.16 [1.06–1.27]) (*p*=.002) and angina pectoris (1.08 [1.02–1.13]) (*p*=.005). Other non-invasive NAFLD scores (FLI, HSI, LAP, HFS, FIB4 and NFS) were also associated with CVDs (Additional Table 3). The utility of LFS as a marker of CVD outcomes (AUC: 0.61–0.66; *p*<.0001) is shown in Additional Table 4. Further subgroup analysis stratified by the presence of diabetes mellitus and body mass index ≥30 kg/m^2^ are shown in Additional Tables 5–9.

**Table 2.** Association of non-alcoholic fatty liver disease liver fat score (LFS) with cardiovascular disease (CVD) outcomes.

	Unadjusted		Model 1		Model 2		
	OR	*p*	OR	*p*	OR	*p*	
CHD	1.29 (1.19 − 1.41)	<.001	1.23 (1.11 − 1.37)	<.001	1.09 (1.03 − 1.15)	.003	
MI	1.23 (1.13 − 1.34)	<.001	1.16 (1.07 − 1.25)	<.001	1.05 (1.00 − 1.11)*	.071	
CHF	1.28 (1.15 − 1.43)	<.001	1.21 (1.07 − 1.37)	.003	1.11 (1.04 − 1.18)	.003	
Stroke	1.18 (1.08 − 1.28)	<.001	1.12 (1.06 − 1.19)	<.001	1.05 (0.97 − 1.13)	.254	
CVD	1.41 (1.31 − 1.51)	<.001	1.36 (1.25 − 1.49)	<.001	1.16 (1.06 − 1.27)	.002	
Angina pectoris	1.24 (1.13 − 1.36)	<.001	1.18 (1.08 − 1.28)	<.001	1.08 (1.02 − 1.13)	.005	

Data are expressed as odds ratio per standard deviation change (95% confidence interval).

OR: odds ratio; CHD: coronary heart disease; MI: myocardial infarction; CHF: congestive heart failure; CVD: composite cardiovascular disease events consisting of CHD, MI, CHF and stroke

Model 1: Adjusted for age, gender and ethnicity.

Model 2: Further adjusted for high-density lipoprotein cholesterol level (mmol/L), smoking status, statin use and aspirin use.

*Due to rounding, odds ratio with 1.00 as lower confidence interval is statistically insignificant.

One-unit SD higher in LFS was associated with increased all-cause mortality and CV mortality (Table 3). After adjusting for age, gender, ethnicity, high-density lipoprotein cholesterol level, smoking status, statin use and aspirin use, LFS was associated with an increased risk of both all-cause and CV mortality with hazard ratio (HR) of 1.10 (95% CI: 1.07–1.13) (*p*<.001) and 1.12 (95% CI: 1.06–1.17) (*p*<.001), respectively. The association of other NAFLD scores (FLI, HSI, LAP, HFS, FIB4 and NFS) with mortality is shown in Additional Table 9.

**Table 3.** Hazard ratios of non-alcoholic fatty liver disease liver fat score (LFS) with all-cause mortality and cardiovascular mortality.

	Unadjusted		Model 1		Model 2	
	HR	*p*	HR	*p*	HR	*p*
All-cause mortality	1.15 (1.11 − 1.18)	<.001	1.10 (1.07 − 1.14)	<.001	1.10 (1.07 − 1.13)	<.001
Cardiovascular mortality	1.15 (1.11 − 1.19)	<.001	1.11 (1.05 − 1.17)	<.001	1.12 (1.06 − 1.17)	<.001

Data are expressed as hazard ratio (HR) per standard deviation (SD) change (95% confidence interval). 15,151 participants are included in mortality study.

HR: hazard ratio

Model 1: Adjusted for age, gender and ethnicity.

Model 2: Further adjusted for high-density lipoprotein cholesterol level (mmol/L), smoking status, statin use and aspirin use.

## 4. Discussion

US NHANES is a large-scale survey in a multi-ethnic, nationally representative and well-characterized study population. Our analysis demonstrated an association of LFS with CVDs, including CHD, CHF and angina pectoris. Our findings are consistent with a previous study that investigated composite CVD events in the NHANES III population [37].

In the setting of a worldwide epidemic of obesity and metabolic syndrome, NAFLD is a growing clinical problem. Non-invasive NAFLD scoring systems are well-validated tools for use in non-hospital settings and have a great advantage over ultrasound at the population level because of expense and convenience. Primary care physicians can screen for fatty liver using routine biochemical tests and anthropometric measurements without expensive equipment and special training.

LFS showed association with most types of CVDs. We have previously reported that LFS is inversely associated with transferrin saturation [17]. Low transferrin saturation is associated with a higher risk of pre-diabetes [38]. We believe that NAFLD may be an early manifestation of the metabolic syndrome; patients with NAFLD may have insulin resistance to some extent and are already at risk of atherosclerotic CVDs before the NAFLD has progressed to liver fibrosis. Evidence from recent Mendelian randomization (MR) studies suggests a casual association between NAFLD and metabolic diseases [39–41]. Genetic predisposition to NAFLD increases the risk of type 2 diabetes mellitus and central obesity while genetically driven type 2 diabetes mellitus promotes the development of NAFLD [39]. Lipid disturbance and changes in adipokines, such as adiponectin, fibroblast growth factor 21 and adipocyte fatty acid-binding protein (A-FABP), also link NAFLD with CVDs [42–44]. A-FABP level is associated with histologically confirmed NAFLD as well as FLI [45,46]. We have previously shown that the A-FABP is a predictor of future CVD events [44]. A-FABP is associated with insulin resistance and vascular inflammation, which may explain its association with CVDs [47]. In a recent MR study, variants in NAFLD susceptibility genes with lipid-lowering effect (PNPLA3: patatin-like phospholipase domain-containing-3; and TM6SF2: transmembrane 6 superfamily member 2) were found to have a protective effect against CHD while variants in another susceptibility gene with lipid-raising effect (GCKR: glucokinase regulatory protein) were associated with CHD. This further suggests that plasma lipid is a putative mediator between NAFLD and CVDs [48,49].

After correction for traditional cardiovascular risk factors, we found that LFS is associated with CHD and angina pectoris, but not MI. Previous studies have shown a significant increase in coronary intima-media thickness and risk of coronary angiogram-determined CHD in NAFLD patients [50]. Progressive atherosclerosis along with coronary artery calcification results in a higher risk of adverse coronary events [20,50]. Meta-analyses have shown a higher risk of MI in subjects with NAFLD [9]. However, a recent large cohort study of Europeans found that the presence of NAFLD was not, after adjustment for conventional CVD risk factors, associated with acute MI [51]. In our analysis, the association of NAFLD with MI and stroke became not significant after adjustment. Thus, NAFLD does not seem to be an independent risk factor for MI and stroke, and it is rather benign unless other CVD risk factors such as diabetes mellitus and hypertension are present. This raises the question as to whether it is worth making the diagnosis. We believe that the use of the score, because it is non-invasive, will identify early NAFLD so that there is early monitoring of CVD risk factors and treating them before CVD complications occur.

We also found that LFS is associated with CHF. There is a close pathological connection between insulin resistance and left ventricle function [52]. Impaired energy metabolism and lipid disturbance alter LV geometry by fibrosis and fatty infiltration [53]. The end-results are ventricular structural and functional abnormalities. It has been well-demonstrated that NAFLD is associated with LV diastolic dysfunction [54] but we are seeing emerging evidence showing its association with impaired systolic function [55]. A recent study detected symptomatic CHF in over one-third of NAFLD patients [53].

Despite being rather benign, NAFLD has been associated with all-cause mortality and the main cause of death, CVD [10,17–20]. Whether there is a direct causal relationship between NAFLD and CV mortality is unclear, but if so, it is likely to be mediated by dyslipidaemia, inflammation, atherosclerosis and ventricular dysfunction. Conventional cardiovascular risk assessment models may not be good at identifying NAFLD-related CVDs. They do not account for insulin resistance, which has an important pathological role in NAFLD. The Framingham Risk Score is known to underestimate the CVD risk in patients with metabolic syndrome . This means that some people whose CVD risks are underestimated are neither on treatment nor under surveillance. The use of a non-invasive score to detect NAFLD may alert the clinician to the need to assess the CV risk of the patient more carefully and act accordingly.

The extent of disease dictates the long-term outcomes of NAFLD. Accumulating evidence shows that more severe NAFLD is associated with an even higher risk of both fatal and non-fatal CVD events [56]. Liver fibrosis biomarkers are also associated with CVDs [29,57,58]. We found that the associations of HFS, FIB4 and NFS with CHD, CHF and CVD were stronger than that of LFS (Table 2, Additional Table 3). This may be explained by the fact that these scores detect patients with more advanced disease and liver fibrosis.

Fibrosis also drives NAFLD-related mortality [59]. Studies of NHANES III database (1988–1994) using FIB4 showed an increase in all-cause and CV mortality in patients with advanced NAFLD [60,61]. Similar observations were also made in biopsy-proven fibrosis and liver fibrosis biomarkers [62]. We also showed that the associations of HFS, FIB4 and NFS with both all-cause and CV mortality are stronger than that of LFS (Table 3, Additional Table 9).

With the emerging evidence showing the link between NAFLD and different types of CVD, it is noteworthy that the European Association for the Study of the Liver, European Association for the Study of Diabetes and European Association for the Study of Obesity clinical practice guidelines recommend comprehensive CVD work-up for all NAFLD patients [63]. The association between NAFLD and CVDs shown in this study supports these guideline recommendations and further emphasizes their importance.

Since measurements of LFS as well as other scores evaluated in this study are simple, convenient and non-invasive, evaluating these biomarkers in the high-risk population could potentially be a cost-effective way for an early identification of NAFLD and its associated cardiovascular morbidities in the setting of primary care. By recognizing cardiovascular risk and intervening early, patients could be protected from future cardiovascular events and CV mortality through timely mitigation of cardiovascular risk.

### 4.1. Limitations

Our analysis is not without limitations. As this study is not a cohort, the temporal relationship between NAFLD and CVD events is unknown. Moreover, records of CVD events were based on self-reported recall, which may be prone to information bias. Although these non-invasive NAFLD scores have been well-validated with either liver biopsy or ultrasonography in previous studies [17, 29–31], ultrasound or FibroScan have not been performed in these NHANES participants to verify the diagnosis of NAFLD. Also, blood samples were only drawn once in each participant, so intraindividual fluctuations in liver enzyme and cholesterol levels over time might not be fully reflected. A transient elevation in liver enzymes levels secondary to alcohol or drug consumption prior to the blood-taking could over-estimate the NAFLD scores.

## 5. Conclusions

With the large study population in NHANES, we demonstrated an association of the LFS with CHD, CHF, angina pectoris, cardiovascular and all-cause mortality. In other words, non-invasive scores of NAFLD severity permit a simple non-invasive “one shot” assessment of both cardio-metabolic and liver-related risks. In view of the high prevalence of NAFLD and the enormous health burden of CVDs, people with NAFLD warrant greater attention to CVD prevention. They should be carefully evaluated for CVDs and the need for preventive treatment, and if they are already on treatment, adherence to treatment and the intensity of treatment should also be reviewed.

## Supplementary Material

Supplemental Material

## Data Availability

The data that support the findings of this study are openly available from the Centres for Disease Control and Prevention at https://www.cdc.gov/nchs/nhanes/index.htm.
