# Supplementary material for: Association between the liver fat score (LFS) and cardiovascular diseases in the national health and nutrition examination survey 1999–2016
Source: Ann Med. 2021 Jun 29;53(1):1067–75. doi: 10.1080/07853890.2021.1943514 (PMC8245099; doi:10.1080/07853890.2021.1943514)
Supplement: Supplemental Material [file IANN_A_1943514_SM2658.docx]

**Supplementary Materials**

**Title: Association between the Liver Fat Score (LFS) and Cardiovascular Diseases in the National Health and Nutrition Examination Survey 1999-2016**

**Authors:** Chun-On Lee, Hang-Long Li, Man-Fung Tsoi, Ching-Lung Cheung, Bernard Man Yung Cheung

**Table of content:**

| Additional Table 1. Formulae of non-invasive non-alcoholic fatty liver disease (NAFLD) scores  Additional Table 2. Means of non-invasive non-alcoholic fatty liver disease (NAFLD) scores  Additional Table 3. Association of other non-invasive non-alcoholic fatty liver disease (NAFLD) scores with cardiovascular disease (CVD) outcomes  Additional Table 4. Utility of non-alcoholic fatty liver disease liver fat score (LFS) as a marker of cardiovascular disease (CVD) outcomes  Additional Table 5. Demographics of participants in subjects with and without diabetes mellitus (DM)  Additional Table 6. Association of non-invasive non-alcoholic fatty liver disease (NAFLD) scores with cardiovascular disease (CVD) outcomes in subjects with and without diabetes mellitus (DM)  Additional Table 7. Demographics of participants in subjects with body mass index (BMI) <30 and ≥30  Additional Table 8. Association of non-invasive non-alcoholic fatty liver disease (NAFLD) scores with cardiovascular disease (CVD) outcomes in subjects with body mass index (BMI) <30 and ≥30  Additional Table 9. Hazard ratios of other non-alcoholic fatty liver disease (NAFLD) scores with all-cause mortality and cardiovascular mortality  Additional Figure 1. Odds ratio plots for associations between cardiovascular disease (CVD) and non-invasive non-alcoholic fatty liver disease (NAFLD) scores | Page 1  Page 2  Page 3-4  Page 5  Page 6  Page 7-8  Page 9  Page 10-11  Page 12  Page 13 |
| --- | --- |

| **Formulae of non-invasive non-alcoholic fatty liver disease (NAFLD) scores** | |
| --- | --- |
| **LFS** | -2.89 + 1.18 x presence of MetS (yes = 1/no = 0) + 0.45 x presence of T2DM (yes = 2/no = 0) + 0.15 x fasting serum insulin (mU/L) + 0.04 x fasting serum AST (U/L) - 0.94 x AST/ALT ratio |
| **FLI** | $\frac{\text{e}^{\text{0.953 ×}\ln\text{(triglyceride, mg/dL) + 0.139 × BMI (kg/}\text{m}^{\text{2}}\text{) + 0.718 × ln (GGT, U/L)}\text{ + 0.053 × waist circumference }\left( \text{cm} \right) \text{- 15.745}}}{\text{1 + }\text{e}^{\text{0.953 ×}\ln\text{(triglyceride, mg/dL) + 0.139×BMI (kg/}\text{m}^{\text{2}}\text{) + 0.718 × ln (GGT, U/L)}\text{ + 0.053 × waist circumference }\left( \text{cm} \right) \text{- 15.745}}}\text{ × 100}$ |
| **HSI** | 8 x ALT/AST ratio + BMI (kg/m^2^) + presence of T2DM (yes = 2/no = 0) + female (yes = 2/ no = 0) |
| **LAP** | [waist circumference (cm) - (65 for men; 58 for women)] x triglycerides (mmol/L) |
| **HFS** | $\frac{1}{\text{1 + }e^{\begin{aligned} (5.390- 0.986 \left( age 45-64 years \right)-1.719 \left( age \geq65 years \right)+0.875 \left( men \right)-0.896 \left( AST 35-69 U/L \right) \\ -2.126 \left( AST \geq70 U/L \right)-0.027 (albumin 4.00-4.49 g/dL)-0.897 \left( albumin <4.00 g/dL \right)-0.899 \\ \left( HOMA 2.00-3.99 without diabetes \right)-1.497 \left( HOMA \geq4.00 without diabetes \right)-2.184 (with \\ diabetes)-0.882 \left( platelet 155-219 x{10}^{9}/L \right)-2.233 (platelet <155 x {10}^{9}/L)) \end{aligned}}}$ |
| **FIB4** | [age (yr) x AST (U/L)] / [platelet count (10^9^/L) x (ALT (U/L))^1/2^] |
| **NFS** | -1.675 + 0.037 x age (years) + 0.094 x BMI (kg/m^2^) + 1.13 x IFG/diabetes (yes = 1/no = 0) + 0.99 x AST/ALT ratio - 0.013 x platelet (10^9^/L) - 0.66 x albumin (g/dL) |

**Additional Table 1. Formulae of non-invasive non-alcoholic fatty liver disease (NAFLD) scores**Abbreviations used in additional table 1: LFS, non-alcoholic fatty liver disease liver fat score; FLI, fatty liver index; HSI, hepatic steatosis index; LAP, lipid accumulation product; HFS, Hepamet fibrosis score; FIB4, fibrosis-4 index; NFS, non-alcoholic fatty liver disease fibrosis score; MetS, metabolic syndrome; mU/L, milliunits per liter; AST, aspartate transaminase level; U/L, units per liter; ALT, alanine aminotransferase level; BMI, body mass index; kg/m^2^, kilograms per square meter; GGT, gamma-glutamyl transferase level; cm, centimeter; T2DM, type 2 diabetes mellitus; mmol/L, millimoles per liter; g/dL, grams per deciliter; HOMA, Homeostatic Model Assessment; L, liter; IFG, impaired fasting glucose.

| **Year** | | **1999-2000** | | **2001-2002** | | **2003-2004** | | **2005-2006** | | **2007-2008** | | **2009-2010** | | **2011-2012** | | **2013-2014** | | **2015-2016** | |  |
| --- | --- | --- | --- | --- | --- | --- | --- | --- | --- | --- | --- | --- | --- | --- | --- | --- | --- | --- | --- | --- |
| **LFS** | | -0.34±0.09 | | -0.39±0.10 | | -0.57±0.09 | | -0.51±0.05 | | -0.36±0.09 | | -0.13±0.06 | | -0.21±0.10 | | -0.29±0.11 | | -0.16±0.09 | *P*<0.001 |  |
| **FLI** | | 48.68±1.79 | | 48.83±0.83 | | 50.9±1.02 | | 51.46±0.98 | | 50.49±0.94 | | 49.58±1.13 | | 50.71±1.36 | | 49.34±0.91 | | 53.44±1.47 | *P*=0.198 |  |
| **HSI*** | | 36.69 (35.85-37.53) | | 36.69 (36.33-37.07) | | 37.14 (36.72-37.57) | | 37.40 (36.97-37.84) | | 36.90 (36.5-37.31) | | 37.10 (36.66-37.55) | | 37.26 (36.72-37.80) | | 37.54 (37.13-37.97) | | 37.95 (37.18-38.74) | *P*=0.040 |  |
| **LAP*** | | 41.23 (37.53-45.28) | | 42.52 (39.75-45.5) | | 45.90 (43.74-48.16) | | 44.25 (41.76-46.89) | | 42.02 (40.02-44.14) | | 40.39 (38.2-42.70) | | 42.84 (39.73-46.2) | | 38.38 (35.98-40.94) | | 43.70 (40.5-47.15) | *P*=0.003 |  |
| **HFS** | | 0.03±0.00 | | 0.04±0.00 | | 0.04±0.00 | | 0.05±0.00 | | 0.05±0.00 | | 0.06±0.00 | | 0.06±0.00 | | 0.06±0.00 | | 0.06±0.00 | *P*<0.001 |  |
| **FIB4*** | | 0.81 (0.76-0.86) | | 0.79 (0.76-0.84) | | 0.84 (0.81-0.87) | | 0.83 (0.78-0.88) | | 0.86 (0.83-0.90) | | 0.98 (0.95-1.01) | | 1.00 (0.95-1.04) | | 0.96 (0.93-1.00) | | 1.02 (0.98-1.07) | *P*<0.001 |  |
| **NFS** | | -2.62±0.06 | | -2.51±0.07 | | -2.29±0.04 | | -2.39±0.08 | | -2.27±0.04 | | -1.88±0.05 | | -1.82±0.06 | | -1.80±0.04 | | -1.73±0.05 | *P*<0.001 |  |

**Additional Table 2. Means of non-invasive non-alcoholic fatty liver disease (NAFLD) scores**

Data are expressed as weighted mean ± standard error, or weighted mean (95% confidence interval).
Abbreviations used in additional table 2: LFS, non-alcoholic fatty liver disease liver fat score; FLI, fatty liver index; HSI, hepatic steatosis index; LAP, lipid accumulation product; HFS, Hepamet fibrosis score; FIB4, fibrosis-4 index; NFS, non-alcoholic fatty liver disease fibrosis score.

^*^Log-transformed score was used.

| **Non-alcoholic fatty liver disease (NAFLD) scores** | | | | | | |  |
| --- | --- | --- | --- | --- | --- | --- | --- |
| **Fatty liver index (FLI)** | | | | | | | |
|  | Unadjusted | | Model 1 | | Model 2 | | |
|  | OR | *P* | OR | *P* | OR | *P* | |
| **CHD** | 1.44 (1.31 – 1.58) | <0.001 | 1.33 (1.19 – 1.49) | <0.001 | 1.06 (0.94 – 1.19) | 0.380 | |
| **MI** | 1.47 (1.35 – 1.62) | <0.001 | 1.36 (1.23 – 1.51) | <0.001 | 1.13 (1.00 – 1.28) | 0.046 | |
| **CHF** | 1.74 (1.52 – 1.99) | <0.001 | 1.72 (1.47 – 2.05) | <0.001 | 1.41 (1.19 – 1.68) | <0.001 | |
| **Stroke** | 1.34 (1.20 – 1.49) | <0.001 | 1.28 (1.13 – 1.45) | <0.001 | 1.11 (0.95 – 1.29) | 0.193 | |
| **CVD** | 1.51 (1.41 – 1.61) | <0.001 | 1.43 (1.32 – 1.55) | <0.001 | 1.19 (1.09 – 1.30) | <0.001 | |
| **Angina pectoris** | 1.58 (1.41 – 1.76) | <0.001 | 1.49 (1.31 – 1.69) | <0.001 | 1.23 (1.08 – 1.41) | 0.002 | |
| **Hepatic steatosis index (HSI)** | | | | | | | |
|  | Unadjusted | | Model 1 | | Model 2 | | |
|  | OR | *P* | OR | *P* | OR | *P* | |
| **CHD** | 1.05 (0.97 – 1.15) | 0.232 | 1.28 (1.16 – 1.42) | <0.001 | 1.02 (0.90 – 1.15) | 0.788 | |
| **MI** | 1.09 (1.00 – 1.18)^*^ | 0.058 | 1.26 (1.14 – 1.40) | <0.001 | 1.06 (0.94 – 1.20) | 0.344 | |
| **CHF** | 1.26 (1.13 – 1.41) | <0.001 | 1.49 (1.30 – 1.70) | <0.001 | 1.27 (1.09 – 1.48) | 0.003 | |
| **Stroke** | 1.14 (1.03 – 1.27) | 0.016 | 1.26 (1.12 – 1.43) | <0.001 | 1.13 (0.98 – 1.29) | 0.098 | |
| **CVD** | 1.17 (1.09 – 1.24) | <0.001 | 1.37 (1.27 – 1.48) | <0.001 | 1.17 (1.07 – 1.27) | <0.001 | |
| **Angina pectoris** | 1.19 (1.08 – 1.30) | <0.001 | 1.37 (1.23 – 1.52) | <0.001 | 1.15 (1.02 – 1.30) | 0.023 | |
| **Lipid accumulation product (LAP)** | | | | | | | |
|  | Unadjusted | | Model 1 | | Model 2 | | |
|  | OR | *P* | OR | *P* | OR | *P* | |
| **CHD** | 1.17 (1.10 – 1.25) | <0.001 | 1.15 (1.07 – 1.24) | <0.001 | 1.09 (1.03 – 1.16) | 0.005 | |
| **MI** | 1.15 (1.09 – 1.22) | <0.001 | 1.13 (1.06 – 1.19) | <0.001 | 1.06 (1.00 – 1.12)^*^ | 0.065 | |
| **CHF** | 1.21 (1.11 – 1.32) | <0.001 | 1.22 (1.11 – 1.35) | <0.001 | 1.14 (1.08 – 1.21) | <0.001 | |
| **Stroke** | 1.15 (1.08 – 1.22) | <0.001 | 1.15 (1.07 – 1.23) | <0.001 | 1.09 (1.02 – 1.17) | 0.018 | |
| **CVD** | 1.22 (1.13 – 1.32) | <0.001 | 1.20 (1.11 – 1.31) | <0.001 | 1.10 (1.04 – 1.16) | 0.001 | |
| **Angina pectoris** | 1.18 (1.10 – 1.27) | <0.001 | 1.16 (1.08 – 1.25) | <0.001 | 1.11 (1.04 – 1.18) | 0.002 | |
| **Hepamet Fibrosis Score (HFS)** | | | | | | | |
|  | Unadjusted | | Model 1 | | Model 2 | | |
|  | OR | *P* | OR | *P* | OR | *P* | |
| **CHD** | 1.54 (1.47-1.63) | <0.001 | 1.25 (1.17-1.33) | <0.001 | 1.15 (1.07-1.24) | <0.001 | |
| **MI** | 1.50 (1.44-1.57) | <0.001 | 1.23 (1.16-1.29) | <0.001 | 1.15 (1.08-1.23) | <0.001 | |
| **CHF** | 1.62 (1.54-1.69) | <0.001 | 1.33 (1.25-1.41) | <0.001 | 1.25 (1.17-1.34) | <0.001 | |
| **Stroke** | 1.47 (1.41-1.54) | <0.001 | 1.16 (1.09-1.22) | <0.001 | 1.09 (1.03-1.17) | <0.001 | |
| **CVD** | 1.67 (1.59-1.75) | <0.001 | 1.29 (1.23-1.35) | <0.001 | 1.21 (1.15-1.27) | <0.001 | |
| **Angina pectoris** | 1.51 (1.44-1.60) | <0.001 | 1.24 (1.16-1.32) | <0.001 | 1.15 (1.06-1.25) | 0.001 | |
| **Fibrosis-4 Index (FIB4)** | | | | | | | |
|  | Unadjusted | | Model 1 | | Model 2 | | |
|  | OR | *P* | OR | *P* | OR | *P* | |
| **CHD** | 1.66 (1.34 – 2.06) | <0.001 | 1.07 (0.99 – 1.15) | 0.106 | 1.11 (1.03 – 1.19) | 0.005 | |
| **MI** | 1.53 (1.22 – 1.90) | <0.001 | 1.05 (0.97 – 1.13) | 0.233 | 1.08 (1.01 – 1.16) | 0.038 | |
| **CHF** | 1.49 (1.15 – 1.92) | 0.003 | 1.10 (1.01 – 1.19) | 0.034 | 1.13 (1.04 – 1.22) | 0.004 | |
| **Stroke** | 1.36 (1.08 – 1.71) | 0.010 | 0.98 (0.90 – 1.06) | 0.616 | 1.00 (0.92 – 1.09) | 0.968 | |
| **CVD** | 1.94 (1.62 – 2.32) | <0.001 | 1.06 (0.98 – 1.16) | 0.162 | 1.10 (1.01 – 1.19) | 0.027 | |
| **Angina pectoris** | 1.40 (1.10 – 1.78) | 0.007 | 1.03 (0.97 – 1.11) | 0.322 | 1.06 (0.99 – 1.14) | 0.113 | |
| **Non-alcoholic fatty liver disease Fibrosis Score (NFS)** | | | | | | | |
|  | Unadjusted | | Model 1 | | Model 2 | | |
|  | OR | *P* | OR | *P* | OR | *P* | |
| **CHD** | 2.79 (2.58-3.02) | <0.001 | 1.62 (1.45-1.82) | <0.001 | 1.34 (1.18-1.53) | <0.001 | |
| **MI** | 2.43 (2.23-2.65) | <0.001 | 1.44 (1.30-1.61) | <0.001 | 1.24 (1.10-1.40) | 0.001 | |
| **CHF** | 2.91 (2.65-3.20) | <0.001 | 1.94 (1.70-2.21) | <0.001 | 1.70 (1.48-1.94) | <0.001 | |
| **Stroke** | 2.29 (2.07-2.53) | <0.001 | 1.39 (1.20-1.62) | <0.001 | 1.26 (1.08-1.47) | 0.003 | |
| **CVD** | 2.78 (2.60-2.98) | <0.001 | 1.62 (1.49-1.77) | <0.001 | 1.41 (1.29-1.55) | <0.001 | |
| **Angina pectoris** | 2.37 (2.15-2.61) | <0.001 | 1.45 (1.25-1.68) | <0.001 | 1.21 (1.04-1.42) | 0.017 | |

**Additional Table 3. Association of other non-alcoholic fatty liver disease (NAFLD) scores with cardiovascular disease (CVD) outcomes**

Data are expressed as odds ratio (OR) per standard deviation change (95% confidence interval).

Abbreviations used in additional table 3: OR, odds ratio; CHD, coronary heart disease; MI, myocardial infarction; CHF, congestive heart failure; CVD, composite cardiovascular disease events consisting of CHD, MI, CHF and stroke.

Model 1: Adjusted for age, gender and ethnicity.

Model 2: Further adjusted for high-density lipoprotein cholesterol level (mmol/L), smoking status, statin use and aspirin use.

^*^Due to rounding, odds ratio with 1.00 as lower confidence interval is statistically insignificant.

|  | **AUC** | ***P*** |
| --- | --- | --- |
| **CHD** | 0.6409 | <0.0001 |
| **MI** | 0.6361 | <0.0001 |
| **CHF** | 0.6555 | <0.0001 |
| **Stroke** | 0.6143 | <0.0001 |
| **CVD** | 0.6417 | <0.0001 |
| **Angina pectoris** | 0.6638 | <0.0001 |

**Additional Table 4. Utility of non-alcoholic fatty liver disease liver fat score (LFS) as a marker of cardiovascular disease (CVD) outcomes**

Data are expressed as area under the ‘receiver operating curve’ (AUC).

Abbreviations used in additional table 4: AUC, area under the ‘receiver operating curve’; CHD, coronary heart disease; MI, myocardial infarction; CHF, congestive heart failure; CVD, composite cardiovascular disease events consisting of CHD, MI, CHF and stroke.

| **Sub-group** | **Without diabetes mellitus (non-DM)** | **With diabetes mellitus (DM)** |  |
| --- | --- | --- | --- |
| **%** | 87.9% | 12.1% |  |
| **Age** | 45.98±0.25 | 59.14±0.36 | *P*<0.001 |
| **Female (%)** | 33,321,921 (51.6%) | 4,246,710 (47.9%) | *P*=0.004 |
| **Ethnicity** |  |  | *P*<0.001 |
| **Mexican American (%)** | 5,040,170 (7.8%) | 779,792 (8.8%) |  |
| **Other Hispanic (%)** | 3,485,425 (5.4%) | 539,322 (6.1%) |  |
| **Non-Hispanic White (%)** | 46,101,792 (71.5%) | 5,651,899 (63.8%) |  |
| **Non-Hispanic Black (%)** | 6,026,278 (9.3%) | 1,237,712 (14.0%) |  |
| **Others (%)** | 3,864,179 (6.0%) | 649,123 (7.3%) |  |
| **HT (%)** | 26,459,789 (41.0%) | 6,846,724 (77.3%) | *P*<0.001 |
| **MetS (%)** | 29,119,907 (45.1%) | 7,583,195 (85.6%) | *P*<0.001 |
| **Medical history** |  |  |  |
| **CHD (%)** | 1,683,358 (2.6%) | 908,477 (10.3%) | *P*<0.001 |
| **MI (%)** | 1,576,297 (2.4%) | 855,995 (9.7%) | *P*<0.001 |
| **CHF (%)** | 966,951 (1.5%) | 668,807 (7.6%) | *P*<0.001 |
| **Stroke (%)** | 1,315,738 (2.0%) | 649,967 (7.3%) | *P*<0.001 |
| **CVD (%)** | 3,786,934 (5.9%) | 1,926,321 (21.7%) | *P*<0.001 |
| **Angina pectoris (%)** | 1,136,456 (1.8%) | 649,617 (7.3%) | *P*<0.001 |
| **Smoker (%)** | 29,404,900 (45.6%) | 4,475,396 (50.5%) | *P*<0.001 |
| **Concomitant medication** |  |  |  |
| **Statin Use (%)** | 7,799,762 (12.1%) | 3,896,886 (44.0%) | *P*<0.001 |
| **Aspirin Use (%)** | 244,952 (0.4%) | 217,918 (2.5%) | *P*<0.001 |
| **Waist circumference (m)** | 0.97±0.00 | 1.10±0.01 | *P*<0.001 |
| **BMI (kg/m^2^)** | 27.49 (27.34-27.64) | 31.8 (31.41-32.18) | *P*<0.001 |
| **Serum HDL cholesterol (mmol/L)^*^** | 1.34 (1.33-1.35) | 1.19 (1.17-1.21) | *P*<0.001 |
| **LFS** | -0.69±0.03 | 2.43±0.12 | *P*<0.001 |
| **FLI** | 47.29±0.41 | 73.31±0.82 | *P*<0.001 |
| **HSI^*^** | 36.45 (36.27-36.63) | 43.24 (42.82-43.67) | *P*<0.001 |
| **LAP^*^** | 38.93 (38.04-39.85) | 76.19 (72.76-79.78) | *P*<0.001 |
| **HFS** | 0.03±0.00 | 0.19±0.00 | *P*<0.001 |
| **FIB4^*^** | 0.87 (0.86-0.88) | 1.19 (1.15-1.23) | *P*<0.001 |
| **NFS** | -2.36±0.02 | -0.31±0.04 | *P*<0.001 |

**Additional Table 5. Demographics of participants in subjects with and without diabetes mellitus (DM)**Data are expressed as the estimated population (weighted percentage), weighted mean ± standard error, or weighted mean (95% confidence interval).
Abbreviations used in additional table 5: HT, hypertension; MetS, metabolic syndrome; CHD, coronary heart disease; MI, myocardial infarction; CHF, congestive heart failure; CVD, composite cardiovascular disease events consisting of CHD, MI, CHF and stroke; m, meter; BMI, body mass index; kg/m^2^, kilogram per square centimeter; HDL, high-density lipoprotein; mmol/L, millimoles per liter; LFS, non-alcoholic fatty liver disease liver fat score; FLI, fatty liver index; HSI, hepatic steatosis index; LAP, lipid accumulation product; HFS, Hepamet fibrosis score; FIB4, fibrosis-4 index; NFS, non-alcoholic fatty liver disease fibrosis score.

*Log-transformed variable was used.

| **Non-alcoholic fatty liver disease (NAFLD) scores** | | | | | | | | | |  |  |
| --- | --- | --- | --- | --- | --- | --- | --- | --- | --- | --- | --- |
| **NAFLD Liver Fat Score (LFS)** | | | | | | | | | |  |  |
|  | **Without diabetes mellitus (non-DM)** | | | | **With diabetes mellitus (DM)** | | | | |  |  |
|  | Unadjusted | | Model 2 | | Unadjusted | | Model 2 | | | |  |
|  | OR | *P* | OR | *P* | OR | *P* | | OR | *P* | | |
| **CHD** | 1.37 (1.23 – 1.49) | <0.001 | 1.12 (0.92 – 1.36) | 0.250 | 1.05 (0.98 – 1.12) | 0.203 | | 1.05 (0.99 – 1.11) | 0.101 | | |
| **MI** | 1.36 (1.27 – 1.47) | <0.001 | 1.23 (1.05 – 1.43) | 0.012 | 0.98 (0.01 – 1.05) | 0.510 | | 0.96 (0.90 – 1.03) | 0.315 | | |
| **CHF** | 1.39 (1.22 – 1.57) | <0.001 | 1.32 (1.04 – 1.69) | 0.027 | 1.05 (1.00 – 1.10)^*^ | 0.061 | | 1.03 (0.98 – 1.09) | 0.269 | | |
| **Stroke** | 1.22 (1.11 – 1.33) | <0.001 | 1.08 (0.89 – 1.31) | 0.435 | 1.00 (0.92 – 1.08) | 0.993 | | 0.98 (0.89 – 1.09) | 0.774 | | |
| **CVD** | 1.40 (1.30 – 1.50) | <0.001 | 1.25 (1.11 – 1.40) | <0.001 | 1.05 (0.99 – 1.12) | 0.088 | | 1.06 (1.01 – 1.12) | 0.029 | | |
| **Angina pectoris** | 1.36 (1.26 – 1.48) | <0.001 | 1.18 (0.98 – 1.42) | 0.076 | 1.02 (0.97 – 1.08) | 0.407 | | 1.02 (0.96 – 1.08) | 0.509 | | |
| **Fatty Liver Index (FLI)** | | | | | | | | | |  |  |
|  | **Without diabetes mellitus (non-DM)** | | | | **With diabetes mellitus (DM)** | | | | |  |  |
|  | Unadjusted | | Model 2 | | Unadjusted | | Model 2 | | | |  |
|  | OR | *P* | OR | *P* | OR | *P* | | OR | *P* | | |
| **CHD** | 1.24 (1.12 – 1.38) | <0.001 | 0.90 (0.79 – 1.03) | 0.132 | 1.14 (0.92 – 1.41) | 0.234 | | 1.34 (1.03 – 1.75) | 0.034 | | |
| **MI** | 1.35 (1.22 – 1.49) | <0.001 | 1.09 (0.95 – 1.25) | 0.212 | 0.99 (0.83 – 1.19) | 0.947 | | 1.03 (0.83 – 1.28) | 0.779 | | |
| **CHF** | 1.47 (1.26 – 1.71) | <0.001 | 1.24 (1.01 – 1.53) | 0.041 | 1.33 (1.07 – 1.64) | 0.010 | | 1.54 (1.17 – 2.03) | 0.003 | | |
| **Stroke** | 1.14 (1.00 – 1.30) | 0.050 | 0.97 (0.81 – 1.18) | 0.783 | 1.14 (0.88 – 1.48) | 0.310 | | 1.33 (0.97 – 1.81) | 0.074 | | |
| **CVD** | 1.31 (1.22 – 1.41) | <0.001 | 1.06 (0.96 – 1.17) | 0.257 | 1.19 (1.01 – 1.39) | 0.036 | | 1.42 (1.18 – 1.71) | <0.001 | | |
| **Angina pectoris** | 1.41 (1.23 – 1.61) | <0.001 | 1.14 (0.97 – 1.35) | 0.113 | 1.12 (0.89 – 1.41) | 0.340 | | 1.21 (0.94 – 1.56) | 0.146 | | |
| **Hepatic Steatosis Index (HSI)** | | | | | | | | | |  |  |
|  | **Without diabetes mellitus (non-DM)** | | | | **With diabetes mellitus (DM)** | | | | |  |  |
|  | Unadjusted | | Model 2 | | Unadjusted | | Model 2 | | | |  |
|  | OR | *P* | OR | *P* | OR | *P* | | OR | *P* | | |
| **CHD** | 0.83 (0.74 – 0.94) | 0.003 | 0.86 (0.73 – 1.00) | 0.057 | 0.85 (0.72 – 0.99) | 0.039 | | 1.12 (0.90 – 1.38) | 0.305 | | |
| **MI** | 0.93 (0.83 – 1.04) | 0.193 | 1.00 (0.85 – 1.17) | 0.956 | 0.78 (0.67 – 0.91) | 0.003 | | 0.95 (0.77 – 1.16) | 0.594 | | |
| **CHF** | 1.00 (0.84 – 1.21) | 0.964 | 1.08 (0.86 – 1.37) | 0.491 | 0.99 (0.85 – 1.15) | 0.855 | | 1.26 (1.00 – 1.59) | 0.050 | | |
| **Stroke** | 0.89 (0.75 – 1.05) | 0.162 | 0.92 (0.74 – 1.14) | 0.430 | 1.05 (0.89 – 1.24) | 0.578 | | 1.35 (1.10 – 1.65) | 0.005 | | |
| **CVD** | 0.96 (0.88 – 1.04) | 0.321 | 1.02 (0.91 – 1.14) | 0.695 | 0.93 (0.83 – 1.05) | 0.240 | | 1.25 (1.09 – 1.44) | 0.002 | | |
| **Angina pectoris** | 0.98 (0.86 – 1.11) | 0.709 | 1.00 (0.85 – 1.18) | 0.978 | 0.94 (0.78 – 1.13) | 0.507 | | 1.15 (0.91 – 1.45) | 0.235 | | |
| **Lipid Accumulation Product (LAP)** | | | | | | | | | |  |  |
|  | **Without diabetes mellitus (non-DM)** | | | | **With diabetes mellitus (DM)** | | | | |  |  |
|  | Unadjusted | | Model 2 | | Unadjusted | | Model 2 | | | |  |
|  | OR | *P* | OR | *P* | OR | *P* | | OR | *P* | | |
| **CHD** | 1.13 (1.06 – 1.20) | <0.001 | 1.02 (0.90 – 1.16) | 0.757 | 1.04 (0.96 – 1.12) | 0.374 | | 1.10 (1.00 – 1.20) | 0.043 | | |
| **MI** | 1.14 (1.07 – 1.21) | <0.001 | 1.06 (0.98 – 1.14) | 0.128 | 0.95 (0.86 – 1.06) | 0.373 | | 0.97 (0.86 – 1.09) | 0.580 | | |
| **CHF** | 1.17 (1.07 – 1.27) | <0.001 | 1.11 (1.02 – 1.20) | 0.017 | 1.08 (1.00 – 1.16) | 0.038 | | 1.11 (1.03 – 1.19) | 0.006 | | |
| **Stroke** | 1.06 (0.99 – 1.14) | 0.096 | 0.90 (0.72 – 1.12) | 0.341 | 1.07 (0.98 – 1.17) | 0.111 | | 1.13 (1.03 – 1.24) | 0.013 | | |
| **CVD** | 1.15 (1.07 – 1.24) | <0.001 | 1.03 (0.95 – 1.11) | 0.471 | 1.04 (0.97 – 1.12) | 0.255 | | 1.10 (1.01 – 1.20) | 0.029 | | |
| **Angina pectoris** | 1.15 (1.07 – 1.23) | <0.001 | 1.07 (0.99 – 1.16) | 0.111 | 1.04 (0.95 – 1.15) | 0.352 | | 1.07 (0.97 – 1.19) | 0.163 | | |
| **Hepamet Fibrosis Score (HFS)** | | | | | | | | | |  |  |
|  | **Without diabetes mellitus (non-DM)** | | | | **With diabetes mellitus (DM)** | | | | |  |  |
|  | Unadjusted | | Model 2 | | Unadjusted | | Model 2 | | | |  |
|  | OR | *P* | OR | *P* | OR | *P* | | OR | *P* | | |
| **CHD** | 1.72 (1.55-1.89) | <0.001 | 1.21 (1.06-1.38) | 0.006 | 1.24 (1.15-1.34) | <0.001 | | 1.12 (1.03-1.22) | 0.011 | | |
| **MI** | 1.63 (1.48-1.80) | <0.001 | 1.13 (0.98-1.32) | 0.100 | 1.20 (1.13-1.28) | <0.001 | | 1.11 (1.03-1.21) | 0.008 | | |
| **CHF** | 1.86 (1.69-2.06) | <0.001 | 1.40 (1.19-1.65) | <0.001 | 1.27 (1.18-1.36) | <0.001 | | 1.12 (1.04-1.21) | 0.004 | | |
| **Stroke** | 1.65 (1.50-1.83) | <0.001 | 1.09 (0.96-1.24) | 0.196 | 1.19 (1.11-1.27) | <0.001 | | 1.02 (0.91-1.15) | 0.738 | | |
| **CVD** | 1.94 (1.76-2.13) | <0.001 | 1.28 (1.16-1.41) | <0.001 | 1.26 (1.19-1.34) | <0.001 | | 1.11 (1.05-1.18) | 0.001 | | |
| **Angina pectoris** | 1.60 (1.45-1.77) | <0.001 | 1.05 (0.87-1.28) | 0.589 | 1.24 (1.16-1.34) | <0.001 | | 1.14 (1.04-1.25) | 0.004 | | |
| **Fibrosis-4 Index (FIB4)** | | | | | | | | | |  |  |
|  | **Without diabetes mellitus (non-DM)** | | | | **With diabetes mellitus (DM)** | | | | |  |  |
|  | Unadjusted | | Model 2 | | Unadjusted | | Model 2 | | | |  |
|  | OR | *P* | OR | *P* | OR | *P* | | OR | *P* | | |
| **CHD** | 1.66 (1.19 – 2.31) | 0.003 | 1.11 (1.03 – 1.20) | 0.009 | 1.38 (1.27 – 1.56) | <0.001 | | 1.10 (0.96 – 1.25) | 0.167 | | |
| **MI** | 1.47 (1.03 – 2.08) | 0.034 | 1.06 (0.97 – 1.15) | 0.193 | 1.36 (1.26 – 1.49) | <0.001 | | 1.14 (1.01 – 1.28) | 0.034 | | |
| **CHF** | 1.42 (0.90 – 2.22) | 0.132 | 1.13 (1.03 – 1.24) | 0.011 | 1.32 (1.21 – 1.46) | <0.001 | | 1.13 (1.03 – 1.25) | 0.015 | | |
| **Stroke** | 1.34 (0.93 – 1.94) | 0.115 | 1.03 (0.94 – 1.14) | 0.510 | 1.18 (1.08 – 1.28) | <0.001 | | 0.93 (0.79 – 1.08) | 0.339 | | |
| **CVD** | 1.96 (1.53 – 2.52) | <0.001 | 1.10 (1.00 – 1.21) | 0.042 | 1.46 (1.30 – 1.65) | <0.001 | | 1.08 (0.97 – 1.21) | 0.157 | | |
| **Angina pectoris** | 1.31 (0.92 – 1.87) | 0.134 | 1.00 (0.91 – 1.11) | 0.923 | 1.31 (1.17 – 1.47) | <0.001 | | 1.14 (0.98 – 1.33) | 0.104 | | |
| **Non-alcoholic fatty liver disease Fibrosis Score (NFS)** | | | | | | | | | |  |  |
|  | **Without diabetes mellitus (non-DM)** | | | | **With diabetes mellitus (DM)** | | | | |  |  |
|  | Unadjusted | | Model 2 | | Unadjusted | | Model 2 | | | |  |
|  | OR | *P* | OR | *P* | OR | *P* | | OR | *P* | | |
| **CHD** | 3.13 (2.79-3.51) | <0.001 | 1.35 (1.12-1.62) | 0.002 | 1.97 (1.64-2.38) | <0.001 | | 1.34 (1.06-1.69) | 0.016 | | |
| **MI** | 2.52 (2.22-2.85) | <0.001 | 1.10 (0.91-1.34) | 0.323 | 1.71 (1.43-2.04) | <0.001 | | 1.20 (0.97-1.49) | 0.102 | | |
| **CHF** | 3.15 (2.69-3.68) | <0.001 | 1.64 (1.28-2.10) | <0.001 | 1.97 (1.61-2.41) | <0.001 | | 1.46 (1.16-1.83) | 0.001 | | |
| **Stroke** | 2.33 (2.01-2.71) | <0.001 | 1.07 (0.87-1.32) | 0.529 | 1.75 (1.42-2.15) | <0.001 | | 1.35 (1.04-1.75) | 0.026 | | |
| **CVD** | 2.91 (2.66-3.20) | <0.001 | 1.19 (1.05-1.35) | 0.006 | 1.98 (1.73-2.28) | <0.001 | | 1.38 (1.17-1.63) | <0.001 | | |
| **Angina pectoris** | 2.39 (2.08-2.74) | <0.001 | 1.02 (0.83-1.24) | 0.871 | 1.68 (1.33-2.12) | <0.001 | | 1.23 (0.92-1.66) | 0.165 | | |

**Additional Table 6. Association of non-invasive non-alcoholic fatty liver disease (NAFLD) scores with cardiovascular disease (CVD) outcomes in subjects with and without diabetes mellitus (DM)**Data are expressed as odds ratio (OR) per standard deviation change (95% confidence interval).
Abbreviations used in additional table 6: OR, odds ratio; CHD, coronary heart disease; MI, myocardial infarction; CHF, congestive heart failure; CVD, composite cardiovascular disease events consisting of CHD, MI, CHF and stroke.
Model 2: Further adjusted for high-density lipoprotein cholesterol level (mmol/L), smoking status, statin use and aspirin use.

*Due to rounding, odds ratio with 1.00 as lower confidence interval is statistically insignificant.

| **Sub-group** | **Body Mass Index (BMI) <30** | **Body Mass Index (BMI) ≥30** |  |
| --- | --- | --- | --- |
| **%** | 65.4% | 34.6% |  |
| **Age** | 47.01±0.30 | 48.63±0.30 | *P*<0.001 |
| **Female (%)** | 23,944,508 (49.9%) | 13,624,123 (53.6%) | *P*<0.001 |
| **Ethnicity** |  |  | *P*<0.001 |
| **Mexican American (%)** | 3,526,997 (7.4%) | 2,292,964 (9.0%) |  |
| **Other Hispanic (%)** | 2,665,015 (5.6%) | 1,359,732 (5.4%) |  |
| **Non-Hispanic White (%)** | 34,396,275 (71.7%) | 17,357,416 (68.3%) |  |
| **Non-Hispanic Black (%)** | 3,874,468 (8.1%) | 3,389,522 (13.3%) |  |
| **Others (%)** | 3,501,457 (7.3%) | 1,011,845 (4.0%) |  |
| **DM (%)** | 3,659,547 (7.6%) | 5,198,300 (20.5%) |  |
| **HT (%)** | 18,055,255 (37.6%) | 15,251,258 (60.0%) | *P*<0.001 |
| **MetS (%)** | 16,643,356 (34.7%) | 20,059,746 (78.9%) | *P*<0.001 |
| **Medical history** |  |  |  |
| **CHD (%)** | 1,532,157 (3.2%) | 1,059,678 (4.2%) | *P*=0.011 |
| **MI (%)** | 1,338,159 (2.8%) | 1,094,133 (4.3%) | *P*<0.001 |
| **CHF (%)** | 872,982 (1.8%) | 762,776 (3.0%) | *P*<0.001 |
| **Stroke (%)** | 1,139,262 (2.4%) | 826,443 (3.3%) | *P*=0.004 |
| **CVD (%)** | 3,238,441 (6.8%) | 2,474,814 (9.7%) | *P*<0.001 |
| **Angina pectoris (%)** | 989,708 (2.1%) | 796,366 (3.1%) | *P*<0.001 |
| **Smoker (%)** | 22,298,445 (46.5%) | 11,581,852 (45.6%) | *P*=0.322 |
| **Concomitant medication** |  |  |  |
| **Statin Use (%)** | 6,650,096 (13.9%) | 5,046,552 (19.9%) | *P*<0.001 |
| **Aspirin Use (%)** | 251,774 (0.5%) | 211,096 (0.8%) | *P*=0.032 |
| **Waist circumference (m)** | 0.90±0.00 | 1.14±0.00 | *P*<0.001 |
| **BMI (kg/m^2^)** | 24.73 (24.64-24.81) | 35.33 (35.15-35.51) | *P*<0.001 |
| **Serum HDL cholesterol (mmol/L)^*^** | 1.39 (1.38-1.41) | 1.20 (1.19-1.21) | *P*<0.001 |
| **LFS** | -1.12±0.03 | 1.20±0.05 | *P*<0.001 |
| **FLI** | 33.04±0.31 | 83.26±0.29 | *P*<0.001 |
| **HSI^*^** | 33.42 (33.30-33.54) | 45.56 (45.34-45.79) | *P*<0.001 |
| **LAP^*^** | 30.47 (29.77-31.20) | 78.09 (76.21-80.02) | *P*<0.001 |
| **HFS** | 0.04±0.00 | 0.08±0.00 | *P*<0.001 |
| **FIB4^*^** | 0.92 (0.90-0.94) | 0.88 (0.86-0.90) | *P*<0.001 |
| **NFS** | -2.47±0.02 | -1.44±0.03 | *P*<0.001 |

**Additional Table 7. Demographics of participants in subjects with body mass index (BMI) <30 and ≥30**Data are expressed as the estimated population (weighted percentage), and weighted mean ± standard error, or weighted mean( 95% confidence interval).
Abbreviations used in additional table 7: DM, diabetes mellitus; HT, hypertension; MetS, metabolic syndrome; CHD, coronary heart disease; MI, myocardial infarction; CHF, congestive heart failure; CVD, composite cardiovascular disease events consisting of CHD, MI, CHF and stroke; m, meter; BMI, body mass index; kg/m^2^, kilogram per square centimeter; HDL, high-density lipoprotein; mmol/L, millimoles per liter; LFS, non-alcoholic fatty liver disease liver fat score; FLI, fatty liver index; HSI, hepatic steatosis index; LAP, lipid accumulation product; HFS, Hepamet fibrosis score; FIB4, fibrosis-4 index; NFS, non-alcoholic fatty liver disease fibrosis score.

*Log-transformed variable was used.

| **Non-alcoholic fatty liver disease (NAFLD) scores** | | | | | | | | | |  |  |
| --- | --- | --- | --- | --- | --- | --- | --- | --- | --- | --- | --- |
| **NAFLD Liver Fat Score (LFS)** | | | | | | | | | |  |  |
|  | **Body mass index (BMI) <30** | | | | **Body mass index (BMI) ≥30** | | | | |  |  |
|  | Unadjusted | | Model 2 | | Unadjusted | | Model 2 | | | |  |
|  | OR | *P* | OR | *P* | OR | *P* | | OR | *P* | | |
| **CHD** | 1.61 (1.42 – 1.83) | <0.001 | 1.14 (0.97 – 1.35) | 0.110 | 1.13 (1.04 – 1.24) | 0.008 | | 1.05 (1.00 – 1.10)^*^ | 0.067 | | |
| **MI** | 1.38 (1.15 – 1.67) | <0.001 | 1.01 (0.91 – 1.13) | 0.799 | 1.10 (1.03 – 1.79) | 0.004 | | 1.02 (0.96 – 1.09) | 0.497 | | |
| **CHF** | 1.37 (1.12 – 1.67) | 0.003 | 1.07 (0.97 – 1.19) | 0.162 | 1.17 (1.01 – 1.36) | 0.041 | | 1.11 (1.04 – 1.19) | 0.003 | | |
| **Stroke** | 1.25 (1.09 – 1.44) | 0.002 | 0.95 (0.84 – 1.06) | 0.354 | 1.11 (1.03 – 1.19) | 0.008 | | 1.08 (1.01 – 1.14) | 0.161 | | |
| **CVD** | 1.73 (1.53 – 1.96) | <0.001 | 1.18 (1.03 – 1.35) | 0.016 | 1.73 (1.53 – 1.96) | <0.001 | | 1.18 (1.03 – 1.35) | 0.016 | | |
| **Angina pectoris** | 1.38 (1.13 – 1.68) | 0.002 | 1.06 (0.98 – 1.16) | 0.152 | 1.12 (1.04 – 1.21) | 0.005 | | 1.07 (1.01 – 1.13) | 0.018 | | |
| **Fatty Liver Index (FLI)** | | | | | | | | | |  |  |
|  | **Body mass index (BMI) <30** | | | | **Body mass index (BMI) ≥30** | | | | |  |  |
|  | Unadjusted | | Model 2 | | Unadjusted | | Model 2 | | | |  |
|  | OR | *P* | OR | *P* | OR | *P* | | OR | *P* | | |
| **CHD** | 1.69 (1.47 – 1.95) | <0.001 | 0.97 (0.80 – 1.18) | 0.784 | 2.34 (1.62 – 3.38) | <0.001 | | 1.67 (1.08 – 2.59) | 0.024 | | |
| **MI** | 1.56 (1.34 – 1.82) | <0.001 | 0.92 (0.75 – 1.12) | 0.393 | 2.05 (1.43 – 2.94) | <0.001 | | 1.40 (0.87 – 2.28) | 0.172 | | |
| **CHF** | 2.01 (1.66 – 2.43) | <0.001 | 1.31 (1.00 – 1.73)^*^ | 0.053 | 3.54 (2.10 – 5.95) | <0.001 | | 3.29 (1.79 – 6.05) | <0.001 | | |
| **Stroke** | 1.46 (1.23 – 1.72) | <0.001 | 1.02 (0.79 – 1.31) | 0.894 | 1.50 (1.00 – 2.27)^*^ | 0.054 | | 1.23 (0.76 – 1.98) | 0.401 | | |
| **CVD** | 1.72 (1.55 – 1.91) | <0.001 | 1.07 (0.93 – 1.25) | 0.345 | 2.12 (1.53 – 2.76) | <0.001 | | 1.65 (1.16 – 2.34) | 0.006 | | |
| **Angina pectoris** | 1.85 (1.56 – 2.19) | <0.001 | 1.14 (0.92 – 1.42) | 0.232 | 2.21 (1.48 – 3.29) | <0.001 | | 1.78 (1.12 – 2.84) | 0.016 | | |
| **Hepatic Steatosis Index (HSI)** | | | | | | | | | |  |  |
|  | **Body mass index (BMI) <30** | | | | **Body mass index (BMI) ≥30** | | | | |  |  |
|  | Unadjusted | | Model 2 | | Unadjusted | | Model 2 | | | |  |
|  | OR | *P* | OR | *P* | OR | *P* | | OR | *P* | | |
| **CHD** | 1.08 (0.87 – 1.34) | 0.496 | 0.83 (0.62 – 1.10) | 0.200 | 0.77 (0.64 – 0.93) | 0.007 | | 1.10 (0.91 – 1.33) | 0.347 | | |
| **MI** | 0.89 (0.72 – 1.10) | 0.291 | 0.70 (0.54 – 0.91) | 0.008 | 0.78 (0.62 – 0.97) | 0.024 | | 1.01 (0.81 – 1.26) | 0.938 | | |
| **CHF** | 1.13 (0.87 – 1.47) | 0.352 | 0.86 (0.61 – 1.20) | 0.373 | 1.14 (0.93 – 1.40) | 0.207 | | 1.54 (1.26 – 1.89) | <0.001 | | |
| **Stroke** | 1.08 (0.82 – 1.42) | 0.584 | 0.93 (0.66 – 1.31) | 0.682 | 1.03 (0.85 – 1.24) | 0.789 | | 1.23 (1.00 – 1.51)^*^ | 0.052 | | |
| **CVD** | 1.14 (0.97 – 1.34) | 0.120 | 0.92 (0.74 – 1.15) | 0.471 | 0.96 (0.85 – 1.08) | 0.520 | | 1.28 (1.12 – 1.46) | <0.001 | | |
| **Angina pectoris** | 1.25 (0.97 – 1.61) | 0.084 | 0.95 (0.71 – 1.28) | 0.757 | 0.94 (0.77 – 1.15) | 0.547 | | 1.19 (0.95 – 1.49) | 0.137 | | |
| **Lipid Accumulation Product (LAP)** | | | | | | | | | |  |  |
|  | **Body mass index (BMI) <30** | | | | **Body mass index (BMI) ≥30** | | | | |  |  |
|  | Unadjusted | | Model 2 | | Unadjusted | | Model 2 | | | |  |
|  | OR | *P* | OR | *P* | OR | *P* | | OR | *P* | | |
| **CHD** | 1.18 (1.07 – 1.29) | 0.001 | 1.05 (0.91 – 1.20) | 0.502 | 1.15 (1.05 – 1.25) | 0.002 | | 1.10 (1.02 – 1.18) | 0.017 | | |
| **MI** | 1.14 (1.05 – 1.23) | 0.001 | 0.91 (0.75 – 1.12) | 0.382 | 1.11 (1.04 – 1.18) | 0.001 | | 1.03 (0.96 – 1.11) | 0.423 | | |
| **CHF** | 1.21 (1.08 – 1.35) | <0.001 | 1.10 (0.99 – 1.22) | 0.068 | 1.16 (1.06 – 1.27) | 0.002 | | 1.13 (1.05 – 1.22) | <0.001 | | |
| **Stroke** | 1.11 (1.04 – 1.20) | 0.003 | 0.82 (0.63 – 1.07) | 0.151 | 1.14 (1.04 – 1.25) | 0.007 | | 1.13 (1.04 – 1.23) | 0.007 | | |
| **CVD** | 1.21 (1.06 – 1.38) | 0.007 | 1.00 (0.91 – 1.11) | 0.939 | 1.16 (1.06 – 1.28) | 0.002 | | 1.10 (1.02 – 1.19) | 0.019 | | |
| **Angina pectoris** | 1.19 (1.07 – 1.32) | 0.001 | 1.07 (1.92 – 1.26) | 0.390 | 1.13 (1.04 – 1.23) | 0.004 | | 1.10 (1.01 – 1.20) | 0.027 | | |
| **Hepamet Fibrosis Score (HFS)** | | | | | | | | | |  |  |
|  | **Body mass index (BMI) <30** | | | | **Body mass index (BMI) ≥30** | | | | |  |  |
|  | Unadjusted | | Model 2 | | Unadjusted | | Model 2 | | | |  |
|  | OR | *P* | OR | *P* | OR | *P* | | OR | *P* | | |
| **CHD** | 1.63 (1.51-1.74) | <0.001 | 1.12 (1.01-1.25) | 0.039 | 1.46 (1.36-1.58) | <0.001 | | 1.19 (1.09-1.31) | <0.001 | | |
| **MI** | 1.56 (1.47-1.65) | <0.001 | 1.09 (0.99-1.20) | 0.079 | 1.43 (1.34-1.52) | <0.001 | | 1.19 (1.10-1.29) | <0.001 | | |
| **CHF** | 1.63 (1.52-1.76) | <0.001 | 1.17 (1.05-1.30) | 0.004 | 1.57 (1.47-1.67) | <0.001 | | 1.32 (1.21-1.43) | <0.001 | | |
| **Stroke** | 1.48 (1.39-1.58) | <0.001 | 0.99 (0.90-1.09) | 0.847 | 1.45 (1.37-1.54) | <0.001 | | 1.19 (1.09-1.30) | <0.001 | | |
| **CVD** | 1.74 (1.62-1.86) | <0.001 | 1.14 (1.05-1.23) | 0.001 | 1.59 (1.48-1.69) | <0.001 | | 1.26 (1.17-1.35) | <0.001 | | |
| **Angina pectoris** | 1.58 (1.46-1.72) | <0.001 | 1.13 (1.00-1.29)^*^ | 0.059 | 1.42 (1.33-1.52) | <0.001 | | 1.16 (1.06-1.27) | 0.002 | | |
| **Fibrosis-4 Index (FIB4)** | | | | | | | | | |  |  |
|  | **Body mass index (BMI) <30** | | | | **Body mass index (BMI) ≥30** | | | | |  |  |
|  | Unadjusted | | Model 2 | | Unadjusted | | Model 2 | | | |  |
|  | OR | *P* | OR | *P* | OR | *P* | | OR | *P* | | |
| **CHD** | 1.59 (1.15 – 2.21) | 0.006 | 1.11 (1.03 – 1.19) | 0.010 | 1.83 (1.56 – 2.14) | <0.001 | | 1.14 (1.00 – 1.30)^*^ | 0.056 | | |
| **MI** | 1.41 (0.99 – 2.01) | 0.060 | 1.03 (0.94 – 1.13) | 0.515 | 1.78 (1.56 – 2.02) | <0.001 | | 1.24 (1.10 – 1.40) | <0.001 | | |
| **CHF** | 1.34 (0.91 – 1.96) | 0.142 | 1.09 (1.02 – 1.17) | 0.013 | 1.79 (1.53 – 2.09) | <0.001 | | 1.30 (1.13 – 1.50) | <0.001 | | |
| **Stroke** | 1.30 (0.94 – 1.80) | 0.117 | 0.99 (0.88 – 1.12) | 0.890 | 1.49 (1.32 – 1.67) | <0.001 | | 1.02 (0.87 – 1.20) | 0.796 | | |
| **CVD** | 1.86 (1.42 – 2.43) | <0.001 | 1.07 (1.00 – 1.15)^*^ | 0.068 | 2.20 (1.96 – 2.47) | <0.001 | | 1.22 (1.09 – 1.36) | <0.001 | | |
| **Angina pectoris** | 1.33 (0.93 – 1.91) | 0.121 | 1.07 (1.00 – 1.15) | 0.046 | 1.55 (1.38 – 1.73) | <0.001 | | 1.05 (0.85 – 1.29) | 0.644 | | |
| **Non-alcoholic fatty liver disease Fibrosis Score (NFS)** | | | | | | | | | |  |  |
|  | **Body mass index (BMI) <30** | | | | **Body mass index (BMI) ≥30** | | | | |  |  |
|  | Unadjusted | | Model 2 | | Unadjusted | | Model 2 | | | |  |
|  | OR | *P* | OR | *P* | OR | *P* | | OR | *P* | | |
| **CHD** | 3.50 (3.08-3.97) | <0.001 | 1.40 (1.14-1.71) | 0.002 | 2.69 (2.36-3.07) | <0.001 | | 1.43 (1.18-1.73) | <0.001 | | |
| **MI** | 2.75 (2.40-3.15) | <0.001 | 1.00 (0.82-1.21) | 0.958 | 2.34 (2.06-2.66) | <0.001 | | 1.40 (1.19-1.65) | <0.001 | | |
| **CHF** | 3.18 (2.74-3.70) | <0.001 | 1.41 (1.11-1.80) | 0.005 | 3.03 (2.60-3.54) | <0.001 | | 2.03 (1.67-2.48) | <0.001 | | |
| **Stroke** | 2.63 (2.24-3.08) | <0.001 | 1.10 (0.86-1.40) | 0.456 | 2.23 (1.88-2.65) | <0.001 | | 1.36 (1.08-1.70) | 0.009 | | |
| **CVD** | 3.24 (2.90-3.61) | <0.001 | 1.24 (1.07-1.44) | 0.004 | 2.73 (2.45-3.04) | <0.001 | | 1.59 (1.38-1.84) | <0.001 | | |
| **Angina pectoris** | 2.72 (2.31-3.21) | <0.001 | 1.05 (0.84-1.30) | 0.673 | 2.25 (1.92-2.63) | <0.001 | | 1.32 (1.05-1.68) | 0.021 | | |

**Additional Table 8. Association of non-invasive non-alcoholic fatty liver disease (NAFLD) scores with cardiovascular disease (CVD) outcomes in subjects with body mass index (BMI) <30 and ≥30**Data are expressed as odds ratio (OR) per standard deviation change (95% confidence interval).
Abbreviations used in additional table 8: OR, odds ratio; CHD, coronary heart disease; MI, myocardial infarction; CHF, congestive heart failure; CVD, composite cardiovascular disease events consisting of CHD, MI, CHF and stroke.
Model 2: Further adjusted for high-density lipoprotein cholesterol level (mmol/L), smoking status, statin use and aspirin use.

*Due to rounding, odds ratio with 1.00 as lower confidence interval is statistically insignificant.

| **Non-alcoholic fatty liver disease (NAFLD) scores** | | | | | | |  |
| --- | --- | --- | --- | --- | --- | --- | --- |
| **Fatty Liver Index (FLI)** | | | | | | | |
|  | Unadjusted | | Model 1 | | Model 2 | | |
|  | HR | *P* | HR | *P* | HR | *P* | |
| **All-cause mortality** | 1.25 (1.18 – 1.33) | <0.001 | 1.07 (1.00 – 1.15) | 0.040 | 1.03 (0.96 – 1.11) | 0.408 | |
| **Cardiovascular mortality** | 1.30 (1.14 – 1.48) | <0.001 | 1.12 (0.96 – 1.30) | 0.141 | 1.06 (0.91 – 1.24) | 0.427 | |
| **Hepatic Steatosis Index (HSI)** | | | | | | | |
|  | Unadjusted | | Model 1 | | Model 2 | | |
|  | HR | *P* | HR | *P* | HR | *P* | |
| **All-cause mortality** | 0.92 (0.87 – 0.98) | 0.010 | 1.00 (0.93 – 1.08) | 0.952 | 0.97 (0.90 – 1.05) | 0.457 | |
| **Cardiovascular mortality** | 0.92 (0.80 – 1.05) | 0.210 | 1.06 (0.91 – 1.24) | 0.432 | 1.02 (0.87 – 1.19) | 0.833 | |
| **Lipid Accumulation Product (LAP)** | | | | | | | |
|  | Unadjusted | | Model 1 | | Model 2 | | |
|  | HR | *P* | HR | *P* | HR | *P* | |
| **All-cause mortality** | 1.10 (1.05 – 1.14) | <0.001 | 1.06 (1.02 – 1.11) | 0.009 | 1.05 (1.00 – 1.10) | 0.043 | |
| **Cardiovascular mortality** | 1.11 (1.06 – 1.16) | <0.001 | 1.09 (1.03 – 1.15) | 0.005 | 1.08 (1.02 – 1.14) | 0.014 | |
| **Hepamet Fibrosis Score (HFS)** | | | | | | | |
|  | Unadjusted | | Model 1 | | Model 2 | | |
|  | HR | *P* | HR | *P* | HR | *P* | |
| **All-cause mortality** | 1.55 (1.51-1.60) | <0.001 | 1.22 (1.18-1.26) | <0.001 | 1.22 (1.18-1.26) | <0.001 | |
| **Cardiovascular mortality** | 1.55 (1.48-1.62) | <0.001 | 1.20 (1.13-1.28) | <0.001 | 1.19 (1.12-1.27) | <0.001 | |
| **Fibrosis-4 Index (FIB4)** | | | | | | | |
|  | Unadjusted | | Model 1 | | Model 2 | | |
|  | HR | *P* | HR | *P* | HR | *P* | |
| **All-cause mortality** | 1.33 (1.21 – 1.47) | <0.001 | 1.19 (1.14 – 1.25) | <0.001 | 1.20 (1.14 – 1.26) | <0.001 | |
| **Cardiovascular mortality** | 1.34 (1.22 – 1.48) | <0.001 | 1.14 (1.02 – 1.28) | 0.021 | 1.15 (1.03 – 1.29) | 0.015 | |
| **Non-alcoholic fatty liver disease Fibrosis Score (NFS)** | | | | | | | |
|  | Unadjusted | | Model 1 | | Model 2 | | |
|  | HR | *P* | HR | *P* | HR | *P* | |
| **All-cause mortality** | 2.51 (2.37-2.66) | <0.001 | 1.30 (1.21-1.40) | <0.001 | 1.30 (1.20-1.40) | <0.001 | |
| **Cardiovascular mortality** | 2.82 (2.49-3.20) | <0.001 | 1.42 (1.17-1.72) | <0.001 | 1.40 (1.15-1.71) | <0.001 | |

**Additional Table 9. Hazard ratios of other non-alcoholic fatty liver disease (NAFLD) scores with all-cause mortality and cardiovascular mortality**

Data are expressed as hazard ratio (HR) per standard deviation (SD) change (95% confidence interval). 15151 participants are included in mortality study.

Abbreviation used in additional table 9: HR, hazard ratio.

Model 1: Adjusted for age, gender and ethnicity.

Model 2: Further adjusted for high-density lipoprotein cholesterol level (mmol/L), smoking status, statin use and aspirin use.

A)

B)

C)

D)

E)

F)

G)


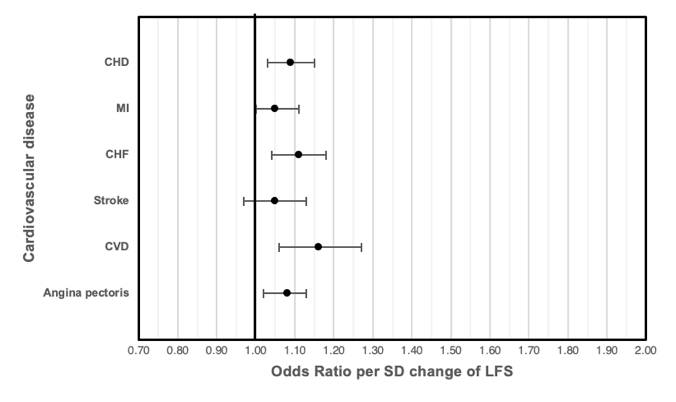

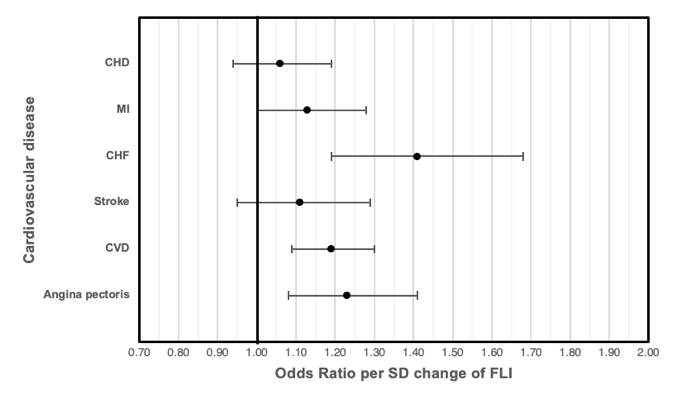

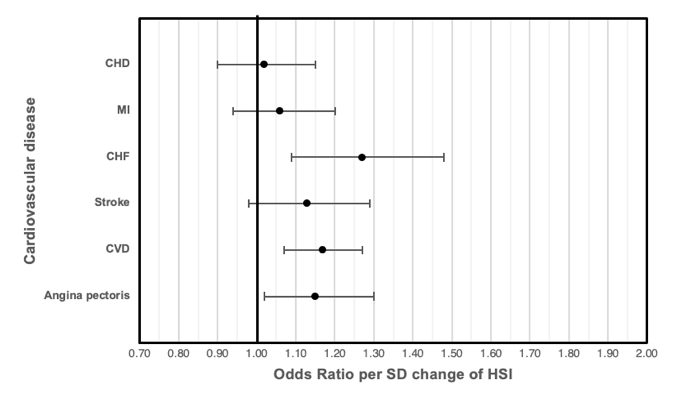

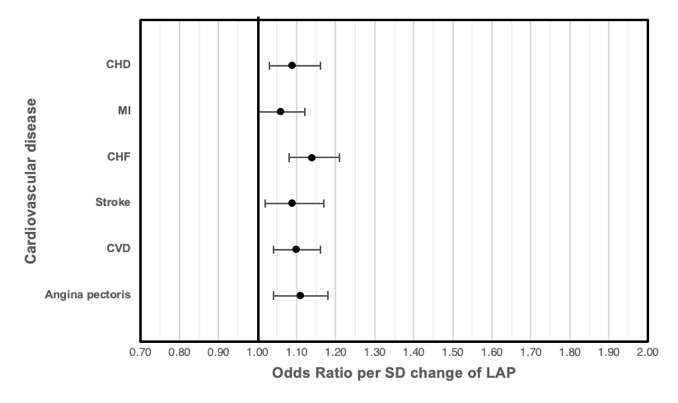

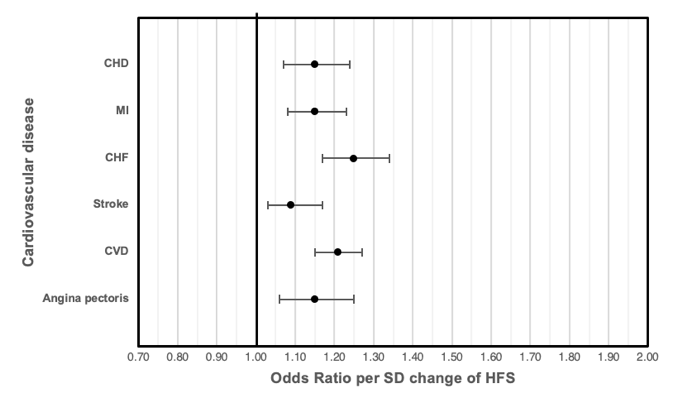

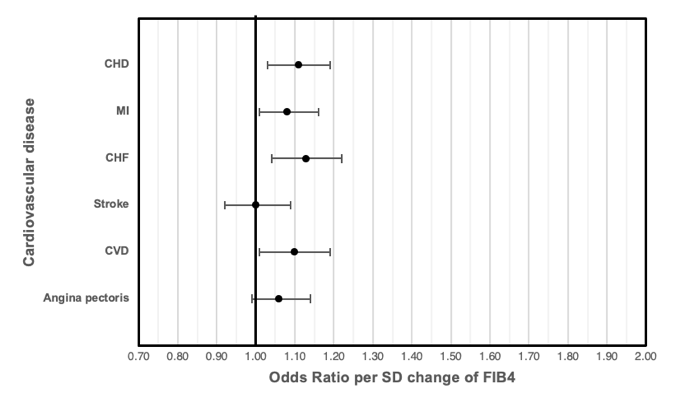

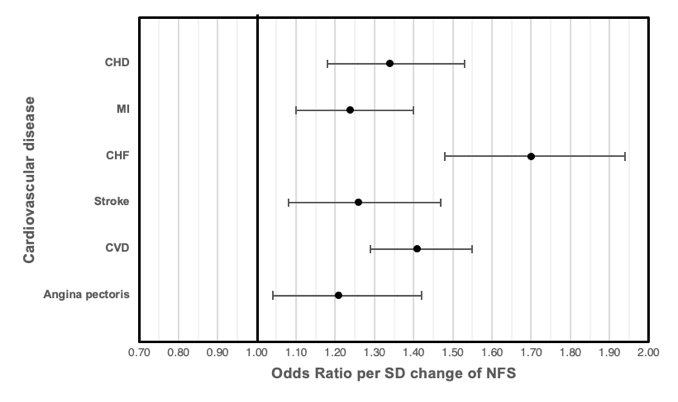


**Additional Figure 1. Odds ratio plots for associations between cardiovascular disease (CVD) and non-invasive non-alcoholic fatty liver disease (NAFLD) scores**A: non-alcoholic fatty liver disease liver fat score (LFS); B: fatty liver index (FLI); C: hepatic steatosis index (HSI); D: lipid accumulation product (LAP); E: HFS, Hepamet fibrosis score; F: fibrosis-4 index (FIB4); G: NFS, non-alcoholic fatty liver disease fibrosis score.
Data are expressed as odds ratio per standard deviation change adjusted for age, gender, ethnicity, high-density lipoprotein cholesterol level, smoking status, statin use and aspirin use with 95% confidence interval.
Abbreviations used in additional figure 1: CHD, coronary heart disease; MI, myocardial infarction; CHF, congestive heart failure; CVD, composite cardiovascular disease events consisting of CHD, MI, CHF and stroke.
